# Effects of Spatial Speech Presentation on Listener Response Strategy for Talker-Identification

**DOI:** 10.3389/fnins.2021.730744

**Published:** 2022-01-28

**Authors:** Stefan Uhrig, Andrew Perkis, Sebastian Möller, U. Peter Svensson, Dawn M. Behne

**Affiliations:** ^1^Department of Electronic Systems, Norwegian University of Science and Technology, Trondheim, Norway; ^2^Quality and Usability Lab, Technische Universität Berlin, Berlin, Germany; ^3^Speech and Language Technology, German Research Center for Artificial Intelligence, Berlin, Germany; ^4^Department of Psychology, Norwegian University of Science and Technology, Trondheim, Norway

**Keywords:** speech perception, spatial auditory cues, talker-identification, voice recognition, sound localization, response strategy, spatial auditory attention, switch costs

## Abstract

This study investigates effects of spatial auditory cues on human listeners' response strategy for identifying two alternately active talkers (“turn-taking” listening scenario). Previous research has demonstrated subjective benefits of audio spatialization with regard to speech intelligibility and talker-identification effort. So far, the deliberate activation of specific perceptual and cognitive processes by listeners to optimize their task performance remained largely unexamined. Spoken sentences selected as stimuli were either clean or degraded due to background noise or bandpass filtering. Stimuli were presented via three horizontally positioned loudspeakers: In a non-spatial mode, both talkers were presented through a central loudspeaker; in a spatial mode, each talker was presented through the central or a talker-specific lateral loudspeaker. Participants identified talkers via speeded keypresses and afterwards provided subjective ratings (speech quality, speech intelligibility, voice similarity, talker-identification effort). In the spatial mode, presentations at lateral loudspeaker locations entailed quicker behavioral responses, which were significantly slower in comparison to a talker-localization task. Under clean speech, response times globally increased in the spatial vs. non-spatial mode (across all locations); these “response time switch costs,” presumably being caused by repeated switching of spatial auditory attention between different locations, diminished under degraded speech. No significant effects of spatialization on subjective ratings were found. The results suggested that when listeners could utilize task-relevant auditory cues about talker location, they continued to rely on voice recognition instead of localization of talker sound sources as primary response strategy. Besides, the presence of speech degradations may have led to increased cognitive control, which in turn compensated for incurring response time switch costs.

## 1. Introduction

The ability of the human auditory system for rapid extraction of spatial auditory cues is thought to facilitate perceptual and cognitive speech processing, especially under adverse and dynamic listening conditions (Zekveld et al., 2014; Koelewijn et al., 2015). Several practical attempts have been made to incorporate advantages of binaural hearing (Blauert, 1997) into the design of spatialized speech displays to counteract degradation factors in speech signal transmission, encompassing application domains like air traffic control (Brungart et al., 2002; Ericson et al., 2004) and audio teleconferencing (Kilgore et al., 2003; Blum et al., 2010; Raake et al., 2010).

Past research usually centered around listening situations involving multiple, simultaneously active talkers. Consequent challenges for auditory information processing are discussed as “cocktail party” problems (Bronkhorst, 2000, 2015), for instance, segregation and streaming of target and masker speech sounds, as well as segmentation and binding to form distinct objects within the auditory scene (Bregman, 1990; Ihlefeld and Shinn-Cunningham, 2008b; Shinn-Cunningham, 2008). The present study addresses another kind of listening situation in dyadic human–human conversation, namely when two talkers take turns in active speaking time (with silence gaps in between). Does auditory information cuing talker location affect behavioral talker-identification (TI) performance in this “turn-taking” listening scenario (Lin and Carlile, 2015, 2019)? If significant effects exist, what are their underlying perceptual and cognitive processes? To what extent are such effects dependent on speech degradations as well as impacting various attributes of subjective listening experience like perceived speech quality, speech intelligibility, or talker-identification effort?^1^

Audio spatialization techniques have been implemented to produce speech signals originating from physical (e.g., presentation through loudspeakers placed in the room) or virtual space (e.g., presentation through stereo headphones, based on head-related transfer functions), which proved advantageous in terms of speech (e.g., word, phrase/sentence) identification performance for simultaneous talkers, speech intelligibility, and listening effort (Yost et al., 1996; Ericson and McKinley, 1997; Nelson et al., 1999; Drullman and Bronkhorst, 2000; Ericson et al., 2004; Kidd et al., 2005b; McAnally and Martin, 2007; Allen et al., 2008; Ihlefeld and Shinn-Cunningham, 2008a,b; Koelewijn et al., 2015). Key influencing factors included the perceived location and relative sound level of talkers as well as listeners' prior knowledge about the task, that is, who will talk, when and where (Brungart et al., 2002; Ericson et al., 2004; Singh et al., 2008; Kitterick et al., 2010; Koelewijn et al., 2015). An in-depth study by Brungart et al. (2005) explored several configurations of auditory and visual cues in a simultaneous two-talker listening situation: presenting each talker through a different, spatially separate loudspeaker had by far the highest impact on word identification performance.

In applied settings, audio spatialization has been recommended as an important design feature of multi-talker speech displays that optimizes its effectiveness without having to attenuate or exclude non-target channels and losing potentially relevant information (Ericson et al., 2004). Related research work suggests strongest effects of spatial auditory information on TI performance if the number of talkers is high, talkers' voices are perceptually similar (e.g., due to same gender of talkers), and quality of transmitted speech is perceived as being low (e.g., due to limited transmission bandwidth) (Blum et al., 2010; Raake et al., 2010; Skowronek and Raake, 2015). Moreover, listening-only test scenarios have proven to be more sensitive to experimental manipulations of audio spatialization (and speech degradation) than conversational test scenarios (Skowronek and Raake, 2015).

Not sufficiently investigated, to date, is the question of how speech stimuli are internally processed by human listeners to achieve fast and accurate TI. Depending on available auditory cues, listeners might develop, combine, or switch between different strategies based on different perceptual and cognitive processes (Allen et al., 2008): During non-spatial speech presentation, TI would have to rely on the recognition of talkers' individual voice characteristics (Best et al., 2018); in the following sections, this cognitive process will be referred to as “voice recognition” (Latinus and Belin, 2011). During spatial speech presentation, TI could instead be based on the localization of active talker sound sources, given that associations between talkers and locations in auditory space are kept unique (Ihlefeld and Shinn-Cunningham, 2008b). As mentioned above, talker-specific spatial cues should become even more relevant when the voices of different talkers are unfamiliar and/or perceptually similar—the latter may also be a consequence of low speech transmission quality in technologically mediated listening situations (Wältermann et al., 2010). Furthermore, TI performance might be differentially affected by different types of speech degradation, like background noise or bandwidth limitation, that impose varying load on human perceptual and cognitive processing (Wickens, 2008) [as can be indicated via methods for continuous physiological recording like pupillometry (Zekveld et al., 2014; Koelewijn et al., 2015) or electroencephalography, EEG (Uhrig et al., 2019a,b)].

The present study deployed a loudspeaker-based test layout to examine listeners' response strategies for behavioral TI during non-spatial and spatial speech presentation modes. A simplified listening scenario with two talkers was realized, wherein only a single talker would be actively speaking at a time. It presumed undisturbed “turn-taking” (i.e., without any instances of talk-over or barge-in) in order to avoid the higher-order acoustic complexity and auditory processing demands of a “cocktail party” scenario involving simultaneous utterances by multiple talkers. Participants were instructed to quickly identify talkers by pushing associated response keys. Task conditions were devised to enable experimental isolation of different kinds of perceptual and cognitive processes (sound source localization, voice recognition).

## 2. Materials and Methods

### 2.1. Participants

The 34 participants recruited for this study were native Norwegian listeners who reported normal hearing as well as normal or corrected-to-normal vision. Data collection, storage, and handling complied with guidelines through the Norwegian Centre for Research Data and with recommendations from the International Committee of Medical Journal Editors (ICMJE). All participants gave their informed consent and received an honorarium at the end of their test sessions, which lasted around one hour.

Due to technical problems during data collection, two participants had to be excluded, leaving a sample size of *N* = 32 (age: *M* = 26.8, *SD* = 5.9, *R* = 19−44 years; 11 female, 21 male; 5 left-handed, 27 right-handed) for further analysis.

### 2.2. Stimuli

The “NB Tale – Speech Database for Norwegian” provided the source stimulus material for the present study. Created by Lingit AS and made publicly available by the National Library of Norway^2^, this database contains a module with audio recordings of sentences, manuscript-read by native talkers from several dialect areas in Norway. Two anonymous male talkers from the Oslo dialect area were chosen for the present study. Twenty sentences had been recorded per talker, which consisted of statements about various neutral topics (based on preceding subjective evaluation by the authors of this paper): Three sentences had the same semantic content for both talkers, whereas the other 17 sentences had different content for each talker. The sentences were of relatively long and varying duration (*M* = 4.9 s, *SD* = 1.5 s, *R* = 2.1− 8.0 s). Presentation of longer, more complex, and variable speech stimuli was deemed a necessary precondition for establishing a more realistic listening situation.

To manipulate speech degradation, all stimuli were presented either as clean, noisy, or filtered versions. Hence, manipulations of two degradation factors, signal-to-noise ratio (SNR) and transmission bandwidth, should entail variation in perceptual quality dimensions of “noisiness” and “coloration,” respectively (Wältermann et al., 2010). The influence of different types of background noise of varying stationarity and informational content on speech quality perception has been investigated before, including pink noise (Leman et al., 2008). Despite traditional telephone bandwidth ranging from 300 to 3,400 Hz (Fernández Gallardo et al., 2012, 2013), a narrower bandpass was chosen in order to provoke a strong enough perceived degradation intensity, similar to recent studies on perceptual discrimination of clean and filtered spoken words (Uhrig et al., 2019a,b). Using the “P.TCA toolbox” for MATLAB software (v. R2018a) (Köster et al., 2015), the clean sentence recordings were impaired along two perceptual dimensions of speech transmission quality (Wältermann et al., 2010) to create degraded stimuli:

- Impairment along the perceptual dimension of “noisiness” was induced by addition of pink noise, aiming at a target SNR of -5 dB, to create noisy stimuli.
- Impairment along the “coloration” dimension was induced by applying a bandpass Butterworth filter, with a low-cutoff frequency of 400 Hz and a high-cutoff frequency of 800 Hz, to create filtered stimuli.

As a final processing step, all 120 generated stimuli (40 clean, 40 noisy, 40 filtered) were normalized to an active speech level of −26 dBov (dBov: decibel relative to the overload point of the digital system) according to ITU-T Recommendation P.56 (2011).

To manipulate audio spatialization, stimuli were presented through different loudspeakers: In the non-spatial mode, stimuli for both talkers were presented through only a single central loudspeaker placed in front of the listener; in spatial modes, stimuli for each talker were presented through either the central or a talker-specific lateral (left, right) loudspeaker, thereby keeping mappings between lateral locations and talkers unique.

### 2.3. Experimental Procedure

Test sessions comprised a talker-identification (TI) task and a talker-localization task, both of which were performed in a quiet, sound-attenuated laboratory room. The order of tasks (identification-localization, localization-identification) was randomized across participants. Table 1 lists the experiment specifications (behavioral tasks, presentation modes, loudspeaker locations, available task-relevant auditory cues, and possible response strategies adopted by listeners).

**Table 1:** Experiment specifications.

**Task**	**Presentation mode**	**Loudspeaker location**	**Cue**	**Strategy**
Talker-identification	Non-spatial_id	Central	Vocal	Voice recognition
	Spatial_id	Central	Vocal	Voice recognition
		Left, Right	Vocal	Voice recognition
			Spatial	Sound localization
Talker-localization	Spatial_loc	Left, Right	Spatial	Sound localization

*Participants carry out behavioral listening tasks (talker-identification, talker-localization) where speech presentation modes (non-spatial_id[entification], spatial_id[entification], spatial_loc[alization]) activate loudspeakers at three spatial locations (left, central, right). The availability of task-relevant auditory cues (vocal, spatial) constraints possible response strategies based on different perceptual and cognitive processes (voice recognition, sound localization)*.

At the beginning of a test session, participants gave their informed consent. They received a print-out information before each task (TI, talker-localization), which instructed them about the two different talkers, the task goals and proper usage of the subjective rating scales (see below). Afterwards, demographic data (age, gender, handedness, vision correction, known hearing problems) were collected.

**Figure 1:**
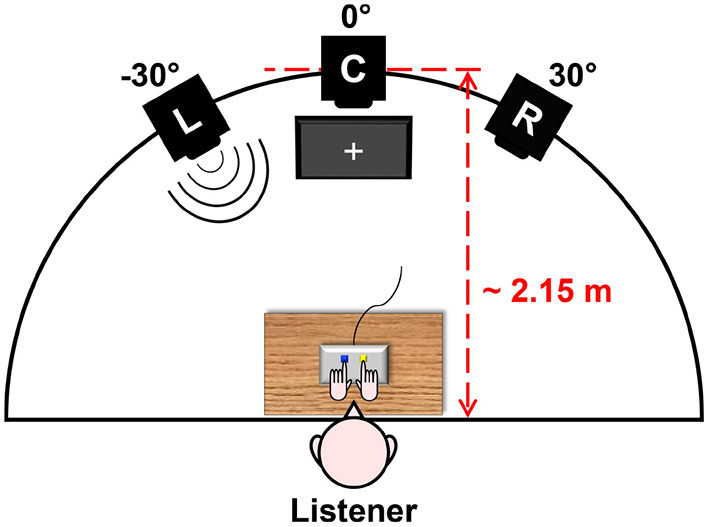
Test layout deployed in the present study. The listener sits at a table, facing an array of three left (L), central (C), and right (R) loudspeakers (L = −30°, C = 0°, R = 30° azimuth) at a distance of approximately 2.15 m. The listener responds to stimuli by pressing keys on a response pad, while fixating a white cross displayed on a monitor screen below C. This figure originally appeared in Uhrig et al. (2020a), copyright 2020, with permission from IEEE.

As illustrated in Figure 1, participants were seated at a small table with a Cedrus RB-740 response pad and a standard computer mouse placed on it. They were facing an array of three Dynaudio BM6A loudspeakers, which were equiangularly separated along the azimuthal direction and elevated approximately at the height of seated listeners' heads. On the floor below the central loudspeaker, a standard computer monitor was positioned. Participants put their left and right index fingers on the left, blue-colored and right, yellow-colored keys of the response pad. By pressing these two response keys, they were able to navigate through task instructions displayed on the monitor screen and respond to presented stimuli. In the task instructions, keys were always referred to by their arbitrarily assigned color (“blue” vs. “yellow”) instead of their direction (“left” vs. “right”) in order to avoid explicit associations of talker locations with keypress responses (Lu and Proctor, 1995). Over the ongoing stimulus presentation phase, participants were asked to fixate a white cross on the monitor to reduce contributions of orienting head movements to binaural hearing (Blauert, 1997).

The TI task was subdivided into six test blocks, one for each experimental condition (see Section 2.4). During a test block, all 40 stimuli (2 talkers × 20 sentences) of a certain *speech degradation* level were presented in series, with an inter-stimulus interval of 1,500 ms, adding a random jitter of either 0, +500, or −500 ms. Silence gaps between spoken sentences were longer than in usual conversational turn-taking [estimated to be around 300–350 ms for conversations in English, see (Lin and Carlile, 2015, 2019)] to ensure that participants would clearly notice when a trial had ended and be ready to respond in the upcoming trial (especially in same-talker trial transitions). To control for general learning and time-on-task effects, the order of conditions was randomized across participants. The order of stimuli was pseudo-randomized across blocks and participants such that only sentences with different semantic content were following each other within the trial sequence. Effects of varying acoustic form of degraded speech stimuli were distinguished from differences in semantic meaning by presenting all sentences in each experimental condition. This counterbalancing of sentence content across conditions is crucial given that variation in speech content has been shown to influence perceived speech quality (Raake, 2002).

In the TI task, participants were instructed to *identify* the current talker after each new stimulus as fast and accurately as possible, by pressing one of the two response keys^3^. In the non-spatial_id mode, stimuli for both talkers were serially presented through the central loudspeaker. In the spatial_id mode, stimuli for a particular talker were presented through the central or one talker-specific lateral (either the left or the right) loudspeaker, with equal probability (i.e., 50% each). Generally, probability of stimulus occurrence was 50% in the center, 25% at the left position, and 25% at the right position (i.e., in sum 50% at any lateral position). The reason for presenting talkers both at lateral positions and at the central position was to ensure that participants had to retain a task set of talker-*identification* throughout the spatial blocks of the TI task (see Table 1). All speech stimuli presented within a block were of the same *speech degradation* level, that is, either clean, noisy, or filtered.

Mappings between talkers and keypress responses for TI (blue vs. yellow) were randomized across blocks. However, in the spatial_id mode, the left and right loudspeaker location was always mapped onto the left (blue) and right (yellow) response key, respectively. Which talker would be presented at which lateral location (i.e., lateral location-talker mappings) was again randomized across blocks. Before starting a new block, participants needed to learn the current talker-response mappings. To accomplish this, they first had to listen (at least once) to a demo stimulus uttered by the particular talker to whom they would respond to with the blue key, and secondly had to listen (at least once) to a demo stimulus uttered by the other talker to respond to with the yellow key. Participants had the option to repeat each demo stimulus as often as they wanted to, for better talker voice memorization before continuing. The two demo stimuli were randomly selected from the current experimental condition and later presented again during the block, yet never occurred as the first stimulus for either talker in the trial sequence.

The talker-localization task consisted of a single test block involving a randomized sequence of clean stimuli, with each talker being randomly presented through the left or right loudspeaker (spatial_loc presentation mode, see Table 1). Participants were asked to indicate the perceived *location* of any active talker as left or right after each new stimulus, as fast and accurately as possible, via left or right keypresses. Thus, here they explicitly engaged in talker-*localization*, contrary to instructions for talker-*identification* in the TI task (see Table 1).

**Figure 2:**
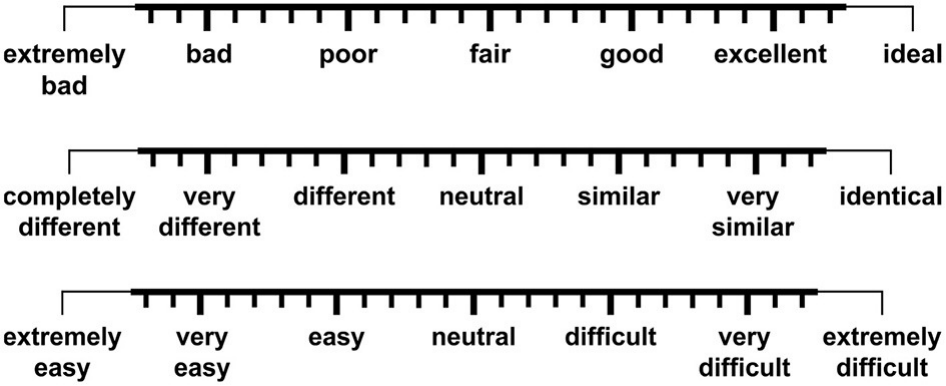
Three continuous rating scales were employed for subjective assessment of perceived speech quality (top scale), speech intelligibility (top scale), voice similarity (middle scale), and talker-identification effort (bottom scale). A seven-point “extended continuous scale” design was implemented in accordance with ITU-T Recommendation P.851 (2003). Another version of this figure originally appeared in Uhrig et al. (2020a), copyright 2020, with permission from IEEE.

At the end of each block continuous rating scales were presented, one after the other, on the monitor screen (for scale design and labelling, see Figure 2). These scales operationalized subjective constructs related to evaluative and task-related attributes of subjective listening experience (speech quality, speech intelligibility, voice similarity, TI effort) (Uhrig et al., 2020a). By using the computer mouse, participants could move a cursor along the full scale range and click at the scale position which in their opinion best described their subjective judgment. The order of scales was randomized across blocks and participants.

Stimulus presentation and data acquisition were executed by means of Psychophysics Toolbox Version 3 (PTB-3)^4^ for MATLAB, running on a Windows-based computer in a control booth adjacent to the laboratory room. Speech files were played back using a high-quality audio interface (Roland UA-1610 Studio-Capture). A sound pressure level meter (Norsonic Nor150) was used to confirm approximately equal loudspeaker levels. The sound pressure level during stimulus presentation was adjusted to be around 65 dB at the listener position, ensuring a comfortable listening level.

### 2.4. Data Analysis

The present study followed a repeated-measures design with two fixed effects, *presentation mode* (non-spatial_id[entification], spatial_id[entification]), and *speech degradation* (clean, noisy, filtered), the full crossing of which resulted in six experimental conditions of the TI task (non-spatial_id/clean, non-spatial_id/noisy, non-spatial_id/filtered, spatial_id/clean, spatial_id/noisy, spatial_id/filtered). One additional *presentation mode* in the talker-localization task (spatial_loc[alization]) served as a talker-localization performance baseline for the ensuing behavioral data analyses (see Section 2.4.2). To further account for variability between participants and speech stimuli, two fully crossed random effects, *subject* (32 levels) and *stimulus* (120 levels), were included in the statistical models.

#### 2.4.1. Rating

For statistical analysis of collected rating data, four repeated-measures analyses of variance (ANOVAs) were computed in R (v. 3.6.1) using the “ez”^5^ package, with *presentation mode* (non-spatial_id, spatial_id) and *speech degradation* (clean, noisy, filtered) as within-subject factors, and rating for each subjective construct (speech quality, speech intelligibility, voice similarity, TI effort) as a dependent variable.

A statistical significance level of α = 0.05 was chosen and Šidák-adjusted for four ANOVAs (α_*SID*_ = 0.013). Generalized eta squared ($\eta_{\text{G}}^{2}$) was computed as an effect size measure. For *post-hoc* comparisons, paired *t*-tests with Holm correction were calculated.

#### 2.4.2. Correct Response Time

To analyze correct response times, only trials with correct keypress responses faster than the 0.95-quantile of the raw response time data series (TI task: *RT*_0.95_ = 1709 ms; talker-localization task: *RT*_0.95_ = 864 ms) were included.

Using “lme4”^6^ and “lmerTest”^7^ packages, linear mixed-effects models (LMEMs) were fitted in R, similar to a previous study (Pals et al., 2015). To mitigate non-normality of their heavily right-skewed distributions, correct response times were log-transformed before entering the LMEMs. The “lmerTest” package provides *p*-values for statistical tests of fixed effects based on Satterthwaite's approximation method (which may give non-integer degrees of freedom) (Kuznetsova et al., 2017).

A statistical significance level of α = 0.05, Šidák-adjusted for five LMEMs (α_*SID*_ = 0.010), was assumed. For *post-hoc* analyses, general linear hypotheses with Holm correction were calculated using the “multcomp”^8^ package.

##### 2.4.2.1. Global Analyses of Presentation Mode

An initial LMEM was computed with *presentation mode* (non-spatial_id, spatial_id) and *speech degradation* (clean, noisy, filtered) as crossed fixed effects, *subject* (32 levels) and *stimulus* (40 levels) as crossed random effects (random intercepts), and correct response time as a dependent variable.

Including only trials from the spatial_id mode, another LMEM was calculated with *speech degradation* and *loudspeaker location* (left, central, right) as crossed fixed effects, *subject* and *stimulus* as crossed random effects (random intercepts), and correct response time as a dependent variable.

##### 2.4.2.2. Local Analyses of Presentation Mode (Lateral/Central Loudspeaker Location)

Two follow-up analysis steps compared behavioral performance at either the center or lateral loudspeaker locations during the spatial_id mode, with spatial_loc and non-spatial_id modes as baselines:

A first analysis contrasted lateral locations (left, right) between the spatial_id mode (TI task) and the spatial_loc mode (talker-localization task). For this purpose, an LMEM was computed with *loudspeaker location* (left, right) and *presentation mode* (spatial_id, spatial_loc) as fixed effects, *subject* and *stimulus* as crossed random effects (random intercepts), and correct response time as a dependent variable.

A second analysis compared average response times between spatial_id and non-spatial_id modes at the central location. Thus, an LMEM was calculated with *presentation mode* (non-spatial_id, spatial_id) and *speech degradation* as fixed effects, *subject* and *stimulus* as crossed random effects (random intercepts), and correct response time as a dependent variable.

##### 2.4.2.3. Analysis of Learning Effects (Within/Across Spatial Blocks)

In a final analysis step, learning effects speeding up behavioral responses within and across the three test blocks employing the spatial_id mode (“spatial blocks”) were analyzed. Prior to trial selection, each spatial block was split into a first and a second half of trials that had presented stimuli through lateral loudspeakers (“lateral trial halfs”).

An LMEM was computed with *loudspeaker location* (left, central, right), *spatial block* (spatial block 1, spatial block 2, spatial block 3) and *lateral trial half* (first trial half, second trial half) as crossed fixed effects, *subject* and *stimulus* as crossed random effects (random intercepts), and correct response time as a dependent variable.

#### 2.4.3. Correct Response Rate

Statistical analyses of correct response rates were carried out using the same R packages described in Section 2.4.2 above. *Correct response rate* was defined as the number of correct keypress responses divided by the total number of keypress responses in a given test block.

A statistical significance level of α = 0.05 was set and Šidák-adjusted for two LMEMs (α_*SID*_ = 0.025). *Post-hoc* analyses involved general linear hypotheses with Holm correction.

##### 2.4.3.1. Global Analysis of Presentation Mode

Selecting only trials from the spatial_id mode, an LMEM was computed with *speech degradation* (clean, noisy, filtered) and *loudspeaker location* (left, central, right) as crossed fixed effects, *subject* (32 levels) as a random effect (random intercept), and correct response rate as a dependent variable.

##### 2.4.3.2. Local Analysis of Presentation Mode (Central Loudspeaker Location)

Focusing solely on the central loudspeaker location, spatial_id and non-spatial_id modes were compared against each other. An LMEM was calculated with *presentation mode* (non-spatial_id, spatial_id), and *speech degradation* as fixed effects, *subject* as a random effect (random intercept), and correct response rate as a dependent variable.

## 3. Expectations

It was anticipated that the two manipulated factors, *presentation mode* (non-spatial_id, spatial_id) and *speech degradation* (clean, noisy, filtered), would influence evaluative and task-related attributes of overall listening experience (speech quality, speech intelligibility, voice similarity, TI effort) (Uhrig et al., 2020a), as well as exert effects on auditory information processing.

Participants should adapt their response strategy to the availability of auditory cues (Kidd et al., 2005a) that are relevant to effectively and efficiently solve their behavioral task goals of quick and accurate TI and talker-localization.

### 3.1. Presentation Mode

During the non-spatial_id mode (TI task), participants would be left only with vocal cues for TI based on voice recognition. However, during the spatial_id mode, performance improvements were expected to depend on *loudspeaker location* (left, central, right): At the central location, again participants would have to rely entirely on voice recognition for identifying different talkers. At lateral (left, right) locations, they would be able to base their response strategy on sound source localization (due to unique lateral location-talker mappings) and ignore the vocal cues, in order to generate quicker and more accurate behavioral responses. Subjectively, this effect should manifest as reduced TI effort for the spatial_id vs. non-spatial_id mode.

Previous studies demonstrated influences of specific target sentence content on word identification performance in multi-talker listening situations (Kidd et al., 2005a; Brungart and Simpson, 2007). In principle, initial portions of talker-specific sentences (see Section 2.2) could offer prosodic [“supra-segmental or phrase-prosody level”; (Fernández Gallardo et al., 2012)] and/or semantic cues (Darwin and Hukin, 2000) to facilitate decision-making about talker identity. Despite this possibility, prosodic/semantic evaluation of each newly occurring stimulus would involve later cognitive processing than rapid sound source localization or voice recognition, thus providing a less efficient alternative/additional response strategy.

Dynamic change in perceived talker location introduces uncertainty in listeners' spatial expectations, ultimately affecting behavioral task performance (Shinn-Cunningham, 2008; Zuanazzi and Noppeney, 2018, 2019). Research on simultaneous multi-talker listening situations demonstrated that prior knowledge about the probability of (change in) talker location leads to improvements in behavioral speech identification (Ericson et al., 2004; Kidd et al., 2005a; Brungart and Simpson, 2007; Singh et al., 2008; Koelewijn et al., 2015). Such improvements are explainable by an anticipatory shift of spatial auditory attention toward the most probable talker location, which in turn enhances information processing within this selected part of the auditory scene. Participants in the present study were not informed in advance about different talker locations or unique lateral location-talker mappings, nor did they receive any feedback on the correctness of their behavioral responses (Kidd et al., 2005a; Best et al., 2018). In spite of this, they would very likely acquire explicit or implicit knowledge about these regularities over the course of the experiment due to incidental statistical/covariation learning (Schuck et al., 2015; Gaschler et al., 2019): It was predicted that within and across spatial blocks participants would utilize talker location cues more and more often, leading to gradually faster behavioral responses at lateral locations.

### 3.2. Speech Degradation

In general, presence of speech degradations should impede TI due to obscuring of individual talkers' voice characteristics. Thus, presentations of degraded (noisy, filtered) speech stimuli would be anticipated to reduce perceived speech quality and speech intelligibility as well as increase voice similarity and TI effort relative to clean stimuli (Leman et al., 2008; Raake et al., 2010; Skowronek and Raake, 2015).

Fernández Gallardo et al. (2012, 2013, 2015) examined human TI performance for spoken words, sentences and paragraph-long speech when being transmitted through wideband and narrowband communication channels. The authors reported notably slower behavioral responses and reduced TI accuracies for narrowband vs. wideband. Studies on speech-in-noise perception established positive relationships between SNR and accuracy of identifying spoken syllables (Kaplan-Neeman et al., 2006), words (Sarampalis et al., 2009) and sentences (Pals et al., 2015), as well as negative relationships between SNR and behavioral response time for identification/recognition of syllables (Kaplan-Neeman et al., 2006), words (Mackersie et al., 1999; Sarampalis et al., 2009) and sentences (Gatehouse and Gordon, 1990; Baer et al., 1993; Houben et al., 2013; Pals et al., 2015); the same relationships were obtained for identification of words within target sentences, being presented concurrently with noise maskers at different SNRs (Ericson and McKinley, 1997; Brungart, 2001; Brungart et al., 2001, 2002; Ericson et al., 2004). Based on this previous evidence, it was expected that behavioral responses to identify single talkers would be generally delayed under degraded (noisy, filtered) vs. clean speech.

Furthermore, this effect of *speech degradation* should probably be more pronounced in the non-spatial_id vs. spatial_id mode, during which participants should rely exclusively on voice characteristics to decide about talker identity.

## 4. Results

### 4.1. Rating

Statistically significant main effects of *speech degradation* resulted for all four subjective constructs: Speech quality [*F*_(2, 62)_ = 357.69, *p* < 0.001, $\eta_{\text{G}}^{2}=0.83$], speech intelligibility [*F*_(2,62)_ = 114.89, *p* < 0.001, $\eta_{\text{G}}^{2}=0.67$], voice similarity [*F*_(2, 62)_ = 21.39, *p* < 0.001, $\eta_{\text{G}}^{2}=0.11$], and TI effort [*F*_(2, 62)_ = 32.54, *p* < 0.001, $\eta_{\text{G}}^{2}=0.18$]. *Post-hoc* pairwise comparisons for clean vs. noisy and clean vs. filtered speech were significant for all constructs (*p* < 0.001); noisy vs. filtered speech showed lower speech quality (*p* < 0.001) and speech intelligibility (*p* < 0.001), and increased TI effort (*p* < 0.01). Neither the main effect of *presentation mode* nor the interaction between the two factors turned out to be statistically significant.

**Figure 3:**
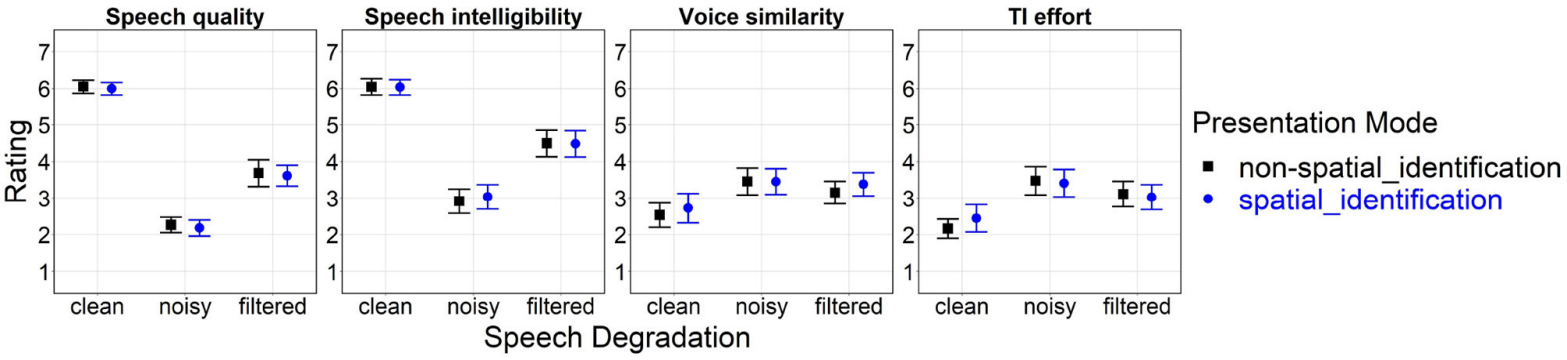
Effects of *presentation mode* and *speech degradation* on rating for evaluative (speech quality, speech intelligibility) and task-related [voice similarity, talker-identification (TI) effort] attributes of overall listening experience. The numeric range of the y-axis (1–7) corresponds to scale labels shown in Figure 2. Error bars represent 95% confidence intervals. Another version of this figure originally appeared in Uhrig et al. (2020a), copyright 2020, with permission from IEEE.

Figure 3 shows arithmetic mean values for effects of *presentation mode* (non-spatial_id, spatial_id) and *speech degradation* (clean, noisy, filtered) on rating for each subjective construct.

### 4.2. Correct Response Time

#### 4.2.1. Global Analyses of Presentation Mode

The initial analysis delivered a significant main effect of *speech degradation* [*F*_(2, 7141.80)_ = 288.71, *p* < 0.001] and a significant interaction between *presentation mode* and *speech degradation* [*F*_(2, 7141.80)_ = 12.87, *p* < 0.001] on correct response time. *Post-hoc* comparisons of the spatial_id mode with the non-spatial_id mode revealed delayed responses for clean speech (*p* < 0.001), faster responses for noisy speech (*p* < 0.001), but no significant difference for filtered speech (*p* = 0.22).

**Figure 4:**
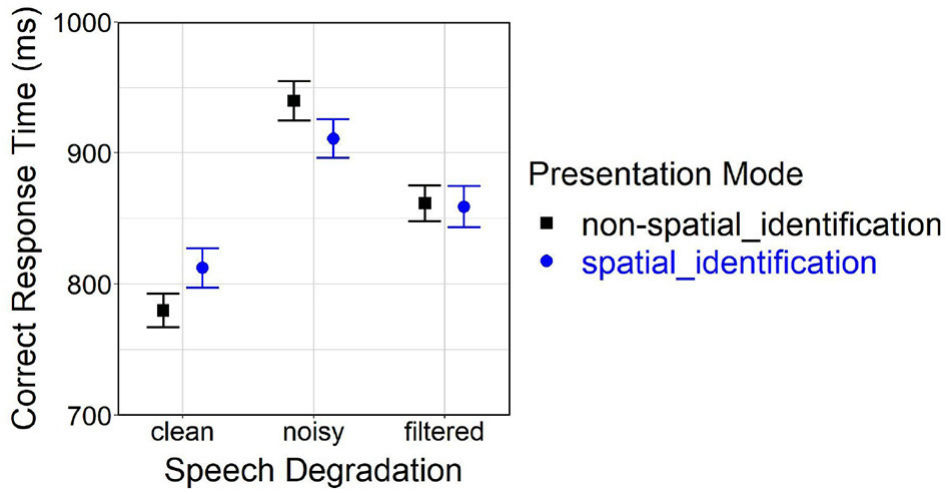
Effects of *presentation mode* and *speech degradation* on correct response time. Error bars represent 95% confidence intervals. Another version of this figure originally appeared in Uhrig (2022), copyright 2021, with permission from Springer.

Figure 4 shows arithmetic mean values for effects of *presentation mode* (non-spatial_id, spatial_id) and *speech degradation* (clean, noisy, filtered) on correct response time.

A follow-up analysis yielded significant main effects of *loudspeaker location* [*F*_(2, 3520.40)_ = 90.56, *p* < 0.001] and *speech degradation* [*F*_(2, 3499.90)_ = 87.16, *p* < 0.001] on correct response time. *Post-hoc* comparisons indicated slower responses at the center vs. lateral (left, right) locations (both *p* < 0.001), but no difference between the left and right location. Significant differences further occurred among all levels of *speech degradation* (all pairs *p* < 0.001): Generally, responses were fastest under clean speech, slower under filtered speech and slowest under noisy speech.

**Figure 5:**
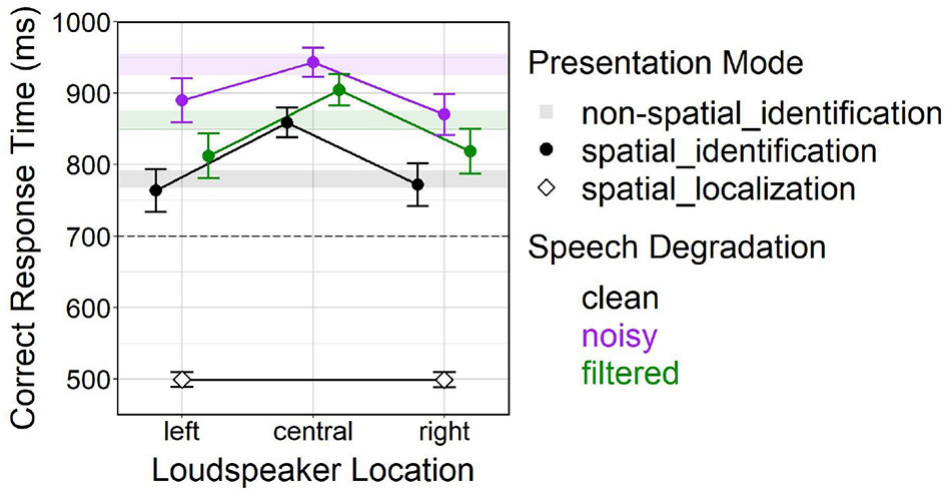
Effects of *speech degradation* and *loudspeaker location* on correct response time in the spatial_id mode. Color-shaded bars represent 95% confidence ranges for the non-spatial_id mode under different *speech degradation* levels (i.e., black bar = non-spatial_id/clean, purple bar = non-spatial_id/noisy, green bar = non-spatial_id/filtered), as depicted in Figure 4. Diamond-shaped points represent the spatial_loc mode (talker-localization task). Error bars represent 95% confidence intervals. The dashed horizontal line at 700 ms marks the lower y-axis limit in Figure 4, for better comparability. Another version of this figure originally appeared in Uhrig (2022), copyright 2021, with permission from Springer.

Figure 5 depicts effects of *speech degradation* and *loudspeaker location* (left, central, right) on correct response time for behavioral TI in the spatial_id mode; the figure also contains confidence ranges of the non-spatial_id mode (bars) and mean values of the spatial_loc mode (diamonds) to serve as baselines, in the latter case for behavioral talker-localization performance.

#### 4.2.2. Local Analyses of Presentation Mode (Lateral/Central Loudspeaker Location)

Two follow-up analyses examined behavioral performance in the spatial_id mode, with spatial_loc and non-spatial_id modes as baselines:

The first analysis confirmed a main effect of *presentation mode* [*F*_(1, 2964.60)_ = 2780.25, *p* < 0.001] on correct response time. Being plainly visible in Figure 5, much faster responses resulted in the spatial_loc vs. spatial_id mode at lateral (left, right) locations (averaged across all levels of *speech degradation*).

The second analysis found significant main effects of *presentation mode* [*F*_(1, 5324.80)_ = 60.75, *p* < 0.001] and *speech degradation* [*F*_(2, 5327.20)_ = 182.01, *p* < 0.001] on correct response time, as well as a significant interaction [*F*_(2, 5326.20)_ = 16.29, *p* < 0.001]. *Post-hoc* comparisons revealed significant differences between all speech degradation levels (all pairwise comparisons *p* < 0.001). Moreover, at the central location, responses were slower in the spatial_id vs. non-spatial_id mode under clean and filtered speech (both *p* < 0.001), but not under noisy speech (*p* = 0.56).

#### 4.2.3. Analysis of Learning Effects (Within/Across Spatial Blocks)

A final analysis showed significant main effects of *loudspeaker location* [*F*_(2, 3511.80)_ = 90.26, *p* < 0.001], *lateral trial half* [*F*_(1, 3499.50)_ = 12.64, *p* < 0.001], and *spatial block* [*F*_(2, 3491.50)_ = 55.18, *p* < 0.001] on correct response time. *Post-hoc* comparisons were again significant between the central location and the lateral (left, right) locations (both *p* < 0.001). A gradual reduction in correct response time was observable both within spatial blocks (first lateral trial half: *M* = 872.92 ms, *SD* = 279.24 ms vs. second: *M* = 848.40 ms, *SD* = 260.62 ms) and across spatial blocks (spatial block 1: *M* = 898.57 ms, *SD* = 280.14 ms vs. 2: *M* = 871.14 ms, *SD* = 269.30 ms vs. 3: *M* = 812.74 ms, *SD* = 253.69 ms; all pairs *p* < 0.001).

**Figure 6:**
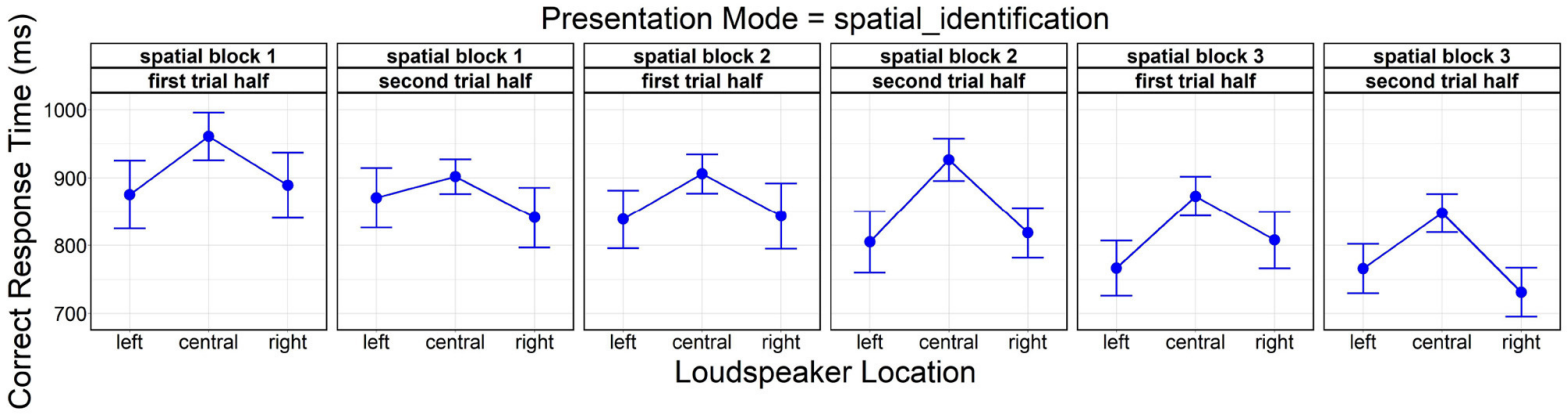
Effects of *loudspeaker location, spatial block* (spatial block 1, spatial block 2, spatial block 3), and *lateral trial half* (first trial half, second trial half) on correct response time. Error bars represent 95% confidence intervals. Another version of this figure originally appeared in Uhrig (2022), copyright 2021, with permission from Springer.

Figure 6 depicts correct response time as a function of *loudspeaker location*, being partitioned into subplots by factor level combinations of *spatial block* and *lateral trial half* to illustrate the temporal development over the succession of spatial blocks.

### 4.3. Correct Response Rate

#### 4.3.1. Global Analyses of Presentation Mode

The initial analysis resulted in a significant main effect of *loudspeaker location* [*F*_(2, 248)_ = 11.76, *p* < 0.001] for correct response rate, also revealing a significant interaction with *speech degradation* [*F*_(4, 248)_ = 3.87, *p* < 0.01]. *Post-hoc* comparisons suggested reduced correct response rates at the central location vs. the lateral (left, right) locations (both *p* < 0.001). The difference between the central location and lateral locations was emerging under clean speech (both *p* < 0.001), but not under degraded (noisy, filtered) speech. This pattern of results is supported by pairwise contrasts between clean and degraded speech that were only significant at the center (clean vs. noisy, *p* < 0.001; clean vs. filtered, *p* = 0.013).

**Figure 7:**
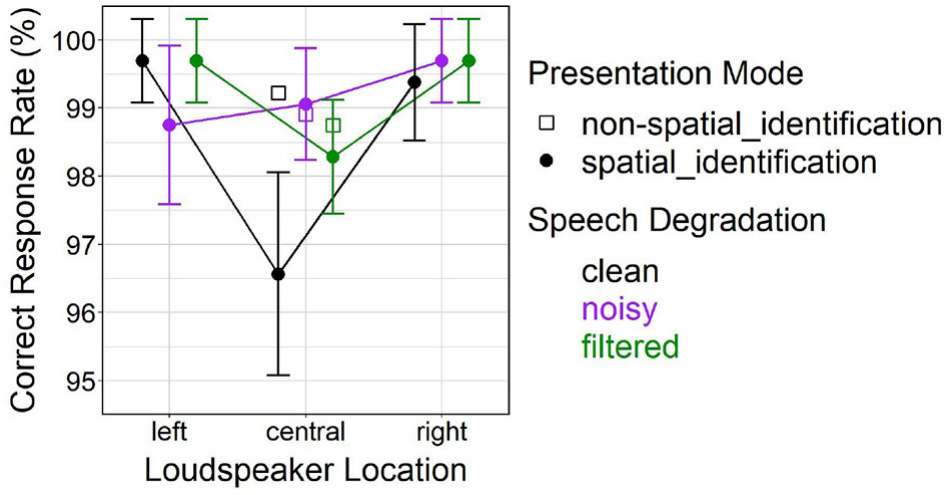
Effects of *presentation mode* and *speech degradation* on correct response rate in the spatial_id mode. Open square-shaped points represent the non-spatial_id mode only (involving presentations only at the central loudspeaker location). Error bars represent 95% confidence intervals.

Figure 7 depicts effects of *speech degradation* and *loudspeaker location* (left, central, right) on correct response rate in the spatial_id mode. The figure further contains mean values in the non-spatial_id mode at the central location (open squares), to aid visual inspection.

#### 4.3.2. Local Analysis of Presentation Mode (Central Loudspeaker Location)

Analyzing only the central location trials demonstrated a significant main effect of *presentation mode* [*F*_(1, 155)_ = 8.13, *p* < 0.01] on correct response rate, and a significant interaction with *speech degradation* [*F*_(2, 155)_ = 6.00, *p* < 0.01]. *Post-hoc* comparisons suggested that average correct response rate was lower for spatial_id vs. non-spatial_id solely for clean speech (*p* < 0.001), as can be seen in Figure 7.

## 5. Discussion

### 5.1. Rating

The rating analysis verified large-sized effects of *speech degradation* on perceived speech quality and speech intelligibility. Regarding the speech degradations induced in the present study, noisy speech impacted those subjective constructs more strongly than filtered speech, when compared to the clean speech reference; this could be attributed to variation in perceived degradation intensity due to different magnitude scaling of each degradation type. On average, the two talkers' voices were perceived to be “different”/“very different” for clean speech, yet slightly more similar for degraded (noisy, filtered) speech, presumably as individual talkers' voice characteristics were masked by added background noise and spectral details were removed by bandpass filtering.

The changes in voice similarity closely corresponded with experienced difficulty of TI, being “easy”/“very easy” under clean speech, but increasing to a small extent under degraded speech (Raake et al., 2010; Zekveld et al., 2014; Skowronek and Raake, 2015). Task difficulty should most probably depend on the amount of allocated information processing resources [i.e., the perceptual-cognitive load (Wickens, 2008); measurable, e.g., by pupillometry or EEG] to discriminate between the two talkers' voices, which was higher for degraded vs. clean speech; better talker voice discriminability would in turn ease TI based on voice recognition. Altogether, experimental manipulation of *speech degradation* could be considered successful, with a weak but nonetheless perceptible impact on TI effort.

No effects of *presentation mode* on any subjective construct turned out to be statistically significant. This stands in direct contrast to a number of previous studies examining “cocktail party” contexts (Raake et al., 2010; Koelewijn et al., 2015; Skowronek and Raake, 2015), which had reported higher perceived speech quality and speech intelligibility as well as reduced talker/speech identification effort following spatial speech presentation. The assessed subjective constructs appeared to be independent of spatialization, at least within the realized, probably less complex “turn-taking” listening scenario. Participants may not have been able to fully exploit talker location cues in the spatial_id mode of the TI task. Possible reasons for this include a lack of prior knowledge about the underlying spatial regularities and/or not having enough exposure time over each spatial block for learning these regularities well enough to noticeably speed up decisions about talker identity. In the future, gathering post-experiment feedback from participants might prove useful to gain better insight into whether they were actually aware of any spatial regularities and intentionally adopted (or refrained from adopting) an alternative localization-based response strategy.

Only half of the trials during the spatial_id mode involved stimulus presentations at talker-specific lateral loudspeaker locations. Participants might have been confused by the random talker location changes between the center and lateral locations, which could have counteracted any advantages of extracted spatial regularities (Uhrig et al., 2020a). Because the spatial_id mode would still demand relatively more effortful voice recognition in half of the trials—namely, when stimuli were presented at the central location—it remains unclear as to whether participants actually experienced a significant overall reduction in task difficulty. Furthermore, with the TI task consistently being judged as “easy,” even under degraded speech, behavioral performance improvements might not have been large enough to be reflected in the subjective ratings, hence constituting a ceiling effect (Best et al., 2018; Uhrig et al., 2020a).

### 5.2. Behavioral Measures (Correct Response Time, Correct Response Rate)

#### 5.2.1. Global Analyses of Presentation Mode

In the initial analysis, significant main effects as well as a significant interaction between *speech degradation* and *presentation mode* on correct response time were observed. Behavioral TI took longer for degraded vs. clean speech, the response delay being more pronounced for noisy vs. filtered speech, which corresponded well with the subjective rating results (see Section 5.1) and past findings (Uhrig et al., 2019a,b). However, contradicting original predictions formulated in Section 3, behavioral responses in the spatial_id vs. non-spatial_id mode were slower for clean, faster for noisy and stayed the same for filtered speech (see Figure 4). This unexpected result pattern could not be easily interpreted without taking the additional factor *loudspeaker location* (left, center, right) into account.

Subsequent analysis consistently showed faster behavioral responses at lateral locations relative to the central location, across all levels of *speech degradation* (see approximately equal slopes of “reverse-V-shaped” lines connecting points within each level of *speech degradation* in Figure 5). In general, behavioral responses were delayed for degraded (noisy, filtered) vs. clean speech. Average correct response rates were very high (around 98–100 %, see Figure 7), remaining constant at lateral locations across the three *speech degradation* levels, only slightly dropping at the center (relative to lateral locations) under clean speech. Interestingly, although at lateral locations the presence of speech degradation had caused a temporal delay in behavioral responses, response accuracy at lateral locations remained unaffected by speech degradation; this fact might be attributable to some form of facilitation of early perceptual and/or late response-related processing, which will be further discussed in Section 5.2.2 below.

#### 5.2.2. Local Analyses of Presentation Mode (Lateral/Central Loudspeaker Location)

The first analysis affirmed behavioral responses at lateral loudspeaker locations to be drastically slower for TI in the spatial_id mode than for talker-localization in the spatial_loc mode (see large deviations between circle- and diamond-shaped points for spatial_id and spatial_loc modes at lateral locations in Figure 5). Therefore, faster behavioral responses at lateral locations (relative to the central location) during the spatial_id mode could not be explained by a spontaneous, full strategy change from voice recognition to sound source localization over the course of a spatial block (Allen et al., 2008; Gaschler et al., 2019). Rather, TI based on voice recognition remained the primary response strategy, but was somehow improved by the additional spatial auditory information. This behavioral response facilitation might possibly originate from automatic, preattentive mechanisms at earlier stages of the auditory processing hierarchy—for instance, improved auditory streaming (Bregman, 1990; Ihlefeld and Shinn-Cunningham, 2008b; Shinn-Cunningham, 2008)—such that the total time needed to reach a behavioral decision on talker identity was slightly shortened.

During all spatial blocks, responses to the talker occurring at the left location corresponded with the left response key and responses to the talker occurring at the right location corresponded with the right response key (see Section 2.3). This constraint controlled for the so called *Simon effect*, describing the phenomenon that behavioral responses are executed faster when located on the same side as their associated stimuli (i.e., being *spatially compatible*; e.g., responding to a stimulus on the left side with the left key) than on the opposite side (i.e., being *spatially incompatible*; e.g., responding to a stimulus on the left side with the right key) (Simon, 1969; Lu and Proctor, 1995). The rationale behind realizing only spatially compatible location-response mappings in the TI and talker-localization tasks was to avoid participant confusion (by spatially incompatible location-response mappings) and instead enable natural directional response tendencies “toward the source of stimulation” (Simon, 1969), hereby improving ecological validity. However, this inevitably led to a confounding influence of *stimulus-response compatibility*: Faster behavioral responses at lateral locations, instead of reflecting facilitated early perceptual processing of spatial information, could also reflect facilitated late response selection. Such automatic response selection might have contributed to the observed “reverse-V-shaped” response time patterns in Figure 5, independently from any hypothetical (partial) adoption of a localization-based response strategy. Future studies might consider isolating possible contributions to TI performance at different perceptual, cognitive, and response-related processing stages (Wickens, 2008).

Surprisingly, differences in correct response time between spatial_id and non-spatial_id modes were also observable at the central loudspeaker location. The second analysis confirmed a response delay in the spatial_id vs. non-spatial_id mode that critically depended on *speech degradation*, being most prominent for clean, less prominent for filtered, and non-significant for noisy speech (see deviations of circle-shaped points from color-shaded bars at the central location in Figure 5). A similar pattern emerged for average correct response rate, where TI performance was reduced in the spatial_id vs. non-spatial_id mode under clean speech, but not under degraded speech (see deviations of circle-shaped points from open square-shaped points at the central location in Figure 7).

Investigations on top-down, intentional control of auditory selective attention (Shinn-Cunningham, 2008) have accrued evidence for a reduction in listening task performance under conditions where the location of a target talker changed vs. stayed constant across trials (Best et al., 2008, 2010; Koch et al., 2011; Lawo et al., 2014; Oberem et al., 2014). Such behavioral performance decrements, known as *switch-costs*, can be caused by repeated switching of selective attention between non-spatial stimulus features (e.g., after changes in target voice gender) (Best et al., 2008; Koch et al., 2011; Koch and Lawo, 2014; Lawo et al., 2014; Lin and Carlile, 2019) as well as between different locations within the auditory scene (Best et al., 2008; Ihlefeld and Shinn-Cunningham, 2008a; Lin and Carlile, 2015) or between ears [e.g., during dichotic listening (Lawo et al., 2014)]. Lin and Carlile (2015) found that unpredictable location changes of target speech (embedded in simultaneous masker speech) decreased performance in memory recall and speech comprehension across successive turn-taking trials, which was attributed to costly switches in spatial attention, disrupted auditory streaming, and increased cognitive processing load. In addition, the authors reported corresponding effects for changes in target voice, which provoked switches in non-spatial selective attention (Lin and Carlile, 2019).

The global slowing of behavioral responses (across all loudspeaker locations) evident for clean speech in the spatial_id vs. non-spatial_id mode, during which talkers changed locations over trials, would support the notion of response time switch costs due to frequent switches in spatial auditory attention (Lawo et al., 2014; Oberem et al., 2014). Another type of response time switch-costs caused by frequent changes in talkers' voice characteristics over trials (Best et al., 2008) could not have systematically affected the results, since talker changes occurred randomly during both spatial_id and non-spatial_id modes. Interestingly, those presumably constant and additive switch-costs manifested most distinctly under clean speech, diminished to some degree under filtered speech and seemingly dissolved under noisy speech. As participants listened to degraded speech, they probably needed to concentrate more intensely on the TI task to ensure an optimal level of performance [i.e., heightened “compensatory effort” (Hockey, 1997)], hereby subjecting their internal information processing to proactive cognitive control mechanisms (Braver, 2012). The incurring switch-costs might therefore have been (partially) compensated, for example, by the preparatory and/or sustained mobilization of spare information processing resources (Chiew and Braver, 2013). On the contrary, while listening to clean speech, processing would either be determined by some degree of reactive cognitive control (Braver, 2012) or be (almost) automated toward the later course of the experiment (Wickens, 2008).

Taking a viewpoint opposite to switches in spatial attention, other studies have emphasized “spatial continuity” as a positive influencing factor on speech identification performance (Best et al., 2008, 2010). However, behavioral responses at the central location were not always faster in the non-spatial_id vs. spatial_id mode (see noisy speech in Figure 5), which would have been anticipated because of refined attentional selection of solely the central location. Thus, spatial continuity did not seem to play a major role in the present TI task.

Likewise, effects of spatial expectation [also: “spatial certainty” (Best et al., 2010)], being caused by varying probability of auditory stimuli occurring at certain locations, could be ruled out as an alternative explanation for the observed result pattern. With a stimulus probability distribution of 25:50:25% over left:central:right loudspeaker locations, the highest chance of stimuli occurring at the central location should have entailed fastest behavioral responses there compared to lateral (left/right) locations (Singh et al., 2008; Zuanazzi and Noppeney, 2018, 2019), which was never the case.

Repeated strategic switching between different perceptual and cognitive processes (i.e., voice recognition vs. sound localization, see Table 1), or between different task sets (TI vs. talker-localization), during a test block can produce another type of response time switch costs (Kiesel et al., 2010). As has already been detailed above, the result patterns shown in Figure 5 imply that no systematic change in primary response strategy (from voice recognition to sound localization) happened during the spatial_id mode (lateral trials) of the TI task—otherwise average performance at lateral locations in the spatial_id mode should be much closer to average performance in the spatial_loc mode (i.e., the slopes of the "reverse-V-shaped" lines in Figure 5 should be much steeper). Therefore, the inferred response time switch costs were not ascribable to strategy or task switching, but rather to frequent shifts in spatial attention focus between the three locations.

Neither the TI task nor the talker-localization task revealed any significant differences between the left and right location, suggesting that ear asymmetries [i.e., differences in processing of auditory information occurrent in the left vs. right hemifield, which arise from hemispheric lateralization (Bolia et al., 2001)] were negligible during the TI and talker-localization tasks.

#### 5.2.3. Analysis of Learning Effects (Within/Across Spatial Blocks)

General learning effects speeding up behavioral responses within and across blocks because of increasing familiarity with the test layout, the current stimulus and task sets were controlled by counterbalancing experimental conditions, location-talker and talker-response mappings across blocks (see Section 2.3). Analysis of learning effects within and across *spatial blocks* implied gradually faster behavioral responses. It further showed the “reverse-V-shaped” response time pattern across *loudspeaker location* as clearly present already in the first lateral trial half of the first spatial block (see first plot from the left in Figure 6); this would substantiate the assumption stated above, namely that automatic perceptual and/or response selection processes may be (jointly) responsible for its emergence, facilitating behavioral responses irrespective of any to-be-acquired (explicit or implicit) knowledge about talker location. Also, since *loudspeaker location* neither interacted with *lateral trial half* nor *spatial block*, the response time benefit at lateral locations did not increase over the course of the TI task (as would be expected from more and more frequent strategic changes from voice recognition to sound localization), which is in line with the conclusion derived above that participants still relied primarily on voice recognition throughout the TI task.

## 6. Conclusion and Outlook

This study investigated the effects of spatial presentation of speech stimuli (spoken sentences) on human talker-identification (TI) performance in a “turn-taking”-like listening situation. In a behavioral TI task, participants extracted available spatial auditory cues during spatial speech presentation to achieve faster responses, which were still considerably slower than responses in a talker-localization task (see Table 1 for experiment specifications, see Section 5.2 for an in-depth behavioral result discussion). Apparently, their primary response strategy remained to be based on the cognitive process of voice recognition.

Moreover, repeated switching of spatial auditory attention between different locations during spatial speech presentation introduced a global delay in behavioral responses. These “response time switch-costs” diminished when speech was degraded by background noise or bandpass filtering, hereby obscuring individual talkers' voice characteristics and increasing subjectively judged talker-identification effort. Internally, this diminishing may have corresponded to greater cognitive control, which in turn activated processes to compensate the switch-costs. A systematic future investigation of the potential role of controlled allocation of information processing resources would necessitate the use of established physiological methods like pupillometry (Zekveld et al., 2014; Koelewijn et al., 2015) or EEG, that enable continuous assessment of the amount of allocated processing resources [i.e., the perceptual-cognitive load (Wickens, 2008)]. Regarding EEG, specifically the P1-N1-P2 complex, the N2, P3a, and P3b components of the event-related brain potential can be considered suitable candidates for neural indication of spatial attention shifts (Getzmann et al., 2015, 2020; Begau et al., 2021) and processing load influenced by varying speech transmission quality (Uhrig et al., 2020b; Uhrig, 2022).

The general lack of a (stronger) impact of spatialization on subjective listening experience and behavioral performance in the present study calls for further exploration. Apparently, constant switching of individual talkers between the central location and talker-specific lateral locations during the spatial presentation mode (TI task) resulted in a more dynamic, less predictable auditory scene, which might have prevented listeners from extracting or (fully) utilizing available spatial auditory cues. It seems intuitive that another spatial mode that *fixes* talkers at separate locations within the auditory scene could alleviate experienced mental effort of TI and speed up behavioral responses. These benefits might be especially pronounced for multimodal, *audio-visual* speech, as it forms the basis of most human-human communication situations. In a recent study by Begau et al. (2021) examining younger and older adults, the presence of visual lip movement in talking faces congruent with auditory speech stimuli was shown to improve perceptual and cognitive speech processing; such audio-visual facilitation effects (manifesting in behavioral and neurophysiological measures) were observable when the target talker remained fixed at the central location, but were cancelled out when the target talker dynamically changed between the central location and lateral locations.

Overall, self-report ratings of speech quality, speech intelligibility and listening effort [as employed, e.g., to subjectively assess speech transmission quality in telephony settings (ITU-T Recommendation P.800, 1996)] seemed to lack sensitivity for detecting more subtle effects of spatialization on TI performance and its interactions with speech degradation (Uhrig et al., 2020a). Follow-up studies might consider adopting a *multi-method* assessment approach, that combines subjective measures with behavioral and (neuro-)physiological measures, in order to achieve highest possible sensitivity as well as convergent validity across multiple levels of analysis. For instance, continuous measurement of behavioral and physiological responses might prove useful to trace performance changes over longer listening episodes (Borowiak et al., 2014).

Viewing the above conclusions from a practical perspective, audio spatialization implemented in modern speech communication systems may not be solely beneficial [as manifested subjectively, e.g., in higher perceived speech quality, improved speech intelligibility/comprehension and reduced mental effort (Baldis, 2001; Kilgore et al., 2003; Raake et al., 2010; Skowronek and Raake, 2015)]. Configurations involving redundant, inconsistent or uncertain mappings between talkers and locations in physical or virtual space could even expend additional information processing resources due to necessary switches in spatial auditory attention. At what scene complexity (e.g., number of spatially separated talkers) and under which contextual listening conditions (e.g., intensity of present speech degradations) such detrimental effects will be strong enough to reach listeners' awareness and/or noticeably reduce their task performance remain open research questions.

## Data Availability Statement

The raw data supporting the conclusions of this article will be made available by the authors, without undue reservation.

## Ethics Statement

Ethical review and approval was not required for the study on human participants in accordance with the local legislation and institutional requirements. The patients/participants provided their written informed consent to participate in this study.

## Author Contributions

SU together with AP, SM, DMB, and UPS developed the concept and methodological approach of the study. SU implemented the experiments in Aura Lab (Dept. of Electronic Systems, NTNU), collected the data, analyzed the data, and drafted the article, which was further revised based on feedback from the coauthors. All authors have read and finally approved the article.

## Funding

This work was supported by the strategic partnership program between the Norwegian University of Science and Technology in Trondheim, Norway, and Technische Universität (TU) Berlin in Berlin, Germany. The experiments reported in this paper have also been part of the corresponding author's (SU's) doctoral thesis, which has been published online in DepositOnce, the repository for research data and publications of TU Berlin (Uhrig, 2021), and in monograph form (Uhrig, 2022).

## Conflict of Interest

The authors declare that the research was conducted in the absence of any commercial or financial relationships that could be construed as a potential conflict of interest.

## Publisher's Note

All claims expressed in this article are solely those of the authors and do not necessarily represent those of their affiliated organizations, or those of the publisher, the editors and the reviewers. Any product that may be evaluated in this article, or claim that may be made by its manufacturer, is not guaranteed or endorsed by the publisher.

## Footnotes

^1^The authors of this article have introduced a detailed Quality of Experience (QoE) model of loudspeaker-based spatial speech presentation in Uhrig et al. (2020a). This QoE model specifies relations between objective degradation factors (quality elements) and subjective attributes of overall listening experience (quality features). It has been validated by analyzing subjective ratings collected during the listening test described in the present study.

^2^ https://www.nb.no/sprakbanken/en/resource-catalogue/oai-nb-no-sbr-31/

^3^A talker-identification task requires discrimination of talker-specific auditory cues, usually pertaining to individual talkers' voice characteristics, as well as retrieval of the (voice) identity category for the current talker from long-term memory (Latinus and Belin, 2011; Best et al., 2018); a talker-verification task requires detection of auditory cues signifying the presence/absence of a particular target talker, for example, as employed in a multi-talker scenario by Drullman and Bronkhorst (2000). It might be argued that identification of the two different talkers in the present study (TI task) is exchangeable for verification of only one of the two talkers, since either way the binary decision outcome would be the same. However, to achieve “true” TI, the identity of *each new talker* must be fully determined, whereas talker-verification is already completed after determining *the particular target talker* as present or absent. Thus, on a functional level, additional internal processes would be involved in the former case.

^4^ http://psychtoolbox.org/

^5^ https://cran.r-project.org/web/packages/ez/

^6^ https://cran.r-project.org/web/packages/lme4/

^7^ https://cran.r-project.org/web/packages/lmerTest/

^8^ https://cran.r-project.org/web/packages/multcomp/
